# Patient safety in marginalised groups: a narrative scoping review

**DOI:** 10.1186/s12939-019-1103-2

**Published:** 2020-02-12

**Authors:** Sudeh Cheraghi-Sohi, Maria Panagioti, Gavin Daker-White, Sally Giles, Lisa Riste, Sue Kirk, Bie Nio Ong, Aaron Poppleton, Stephen Campbell, Caroline Sanders

**Affiliations:** 1 NIHR Greater Manchester Patient Safety Translational Research Centre, The University of Manchester, Williamson Building, Oxford Rd, Manchester, M13 9PL, England; 2 Centre for Primary Care, The University of Manchester, Williamson Building, Oxford Rd, Manchester, M13 9PL England; 3 Keele University, Citylabs, Nelson St, Manchester, M13 9NQ England; 4 NIHR School for Primary Care Research, Citylabs, Nelson St, Manchester, M13 9NQ England; 5 Health Innvoation Manchester, Citylabs, Nelson St, Manchester, M13 9NQ England

**Keywords:** Patient safety, Marginalised groups, Contributory factors, Scoping review

## Abstract

**Background:**

Marginalised groups (‘populations outside of mainstream society’) experience severe health inequities, as well as increased risk of experiencing patient safety incidents. To date however no review exists to identify, map and analyse the literature in this area in order to understand 1) which marginalised groups have been studied in terms of patient safety research, 2) what the particular patient safety issues are for such groups and 3) what contributes to or is associated with these safety issues arising.

**Methods:**

Scoping review. Systematic searches were performed across six electronic databases in September 2019. The time frame for searches of the respective databases was from the year 2000 until present day.

**Results:**

The searches yielded 3346 articles, and 67 articles were included. Patient safety issues were identified for fourteen different marginalised patient groups across all studies, with 69% (*n* = 46) of the studies focused on four patient groups: ethnic minority groups, frail elderly populations, care home residents and low socio-economic status. Twelve separate patient safety issues were classified. Just over half of the studies focused on three issues represented in the patient safety literature, and in order of frequency were: medication safety, adverse outcomes and near misses. In total, 157 individual contributing or associated factors were identified and mapped to one of seven different factor types from the Framework of Contributory Factors Influencing Clinical Practice within the London Protocol. Patient safety issues were mostly multifactorial in origin including patient factors, health provider factors and health care system factors.

**Conclusions:**

This review highlights that marginalised patient groups are vulnerable to experiencing a variety patient safety issues and points to a number of gaps. The findings indicate the need for further research to understand the intersectional nature of marginalisation and the multi-dimensional nature of patient safety issues, for groups that have been under-researched, including those with mental health problems, communication and cognitive impairments. Such understanding provides a basis for working collaboratively to co-design training, services and/or interventions designed to remove or at the very least minimise these increased risks.

**Trial registration:**

Not applicable for a scoping review.

## Background

Improving patient safety is at the forefront of healthcare policy and practice across the globe [1] but may be especially challenging for marginalised groups of patients [2–4]. The European Network for social inclusion and health defines marginalisation as the “position of individuals, groups or populations outside of ‘mainstream society’”) [5]. Marginalised patients experience severe health inequities which can result in poorer health status, higher premature morbidity and increased risk for patient safety incidents in comparison to the general population [6] [2–4]. There are several reasons underlying these poor health care outcomes among marginalised patients. At the macro-level for example, marginalised people may have no voice on healthcare policy planning and/or resource allocation because they are “*systemically excluded from national or international policy making forums”* [5] [7]. At the meso-level, poor or non-inclusive organisational service designs can lead to gaps in service provision for marginalised patients [3]. Finally, at the micro-level, marginalised people may experience barriers to communication regarding their health care needs and treatment due to impairment or personal context (e.g. language barriers or sensory, learning or age related disability) [8, 9] or as a consequence of perceived [10] or actual stigma enacted (e.g. labelling of some homeless patients as ‘difficult’ leading to barriers in accessing care) [3, 11].

Although published reviews have sought to capture the nature, causes and consequences of patient safety incidents in various settings [12, 13], to our knowledge, none have specifically focused on marginalised populations. A scoping review is particularly suited to when the aim is to identify and map out the literature as opposed to a systematic review, which typically aims to responds to a very specific well defined research questions for a specific patient group [14]. We therefore chose the scoping review approach in order to determine the range of patient safety issues and in which types of marginalised patient groups. In order to be inclusive when mapping this potentially diverse literature, we also chose the broader definition of ‘patient safety issues’ [15] as opposed to a specific patient safety incident, to enable consideration of wider underlying circumstance and complexities for patients from marginalised groups as opposed to those from the general population.

This scoping review examines the range of patient safety issues for people considered to be marginalised. Our four main aims were: 1) to identify which marginalised patient groups have been studied in terms of patient safety research, 2) to understand what the particular patient safety issues are for these groups and 3) what contributes to the safety issues arising.

## Methods

This scoping review was conducted in accordance with the guidance for conducting systematic scoping reviews [16].

### Definitions

In the absence of an identifiable agreed definition within the literature, we chose as stated above, the European Network for social inclusion and health’s definition of marginalisation, which simply states that marginalisation is the “position of individuals, groups or populations outside of ‘mainstream society” [5]. The definition is broad and reflects the fact that marginalisation in an umbrella term. Marginalised people however can be grouped due to them sharing common features or outcomes (e.g. reduced access to health services) as a result of their marginalisation, but may have other differing attributes (e.g. ethnicity, disability etc.) which lead or have led to their marginalisation. We do hypothesise however that marginalised groups may experience negative consequences or disparities in patient safety as a result of their marginalisation. Consequently, we also included studies utilising the terms ‘seldom heard,’ ‘hard to read’ and vulnerable groups.’ The inclusion of these terms reflects the fact that they have also been used in the literature to represent the same groups designated as marginalised elsewhere in the literature. Hard-to-reach, for example, is a term cited by National Health Service (NHS) reports in the UK [17]. These reports acknowledge that certain groups are marginalised from services and therefore ‘harder to reach’ for health services whose goal is to provide appropriate and equitable health care for all populations. ‘Seldom heard’ groups have been defined as groups who may experience barriers to accessing services or are under-represented in healthcare decision making [18, 19]. Finally, vulnerability has been defined as “susceptibility to any kind of harm whether physical, moral or spiritual, at the hands of an agent or agency” [20], a factor which “… needs to be recognised and negotiated in health care transactions.” [21] . The Organisation for Economic Co-operation and Development (OECD) report into integrating Social Services for Vulnerable Groups defines ‘vulnerable populations’ as “people or households who live in poverty, or who are confronted with life situations that increase the likelihood of extreme forms of poverty [22]. These populations often face multiple risks and may require a range of services, from low-cost interventions such as food parcels, to more costly interventions such as housing, or mental or physical health care.” Vulnerability can be identified as occurring as a result of one or more social, structural, situational or other causes. Such definitions and causes clearly have significant overlap with the definitions for marginalised groups and have clear applications to patient safety within a healthcare context.

### Patient and public involvement

We worked with our patient-research partners in one of our departmental patient and public involvement (PPI) groups in the design of the study. Specific suggestions were made by the PPI groups and added to the protocol such as additional terms (e.g. care leaver) for the search strategy.

### Data sources and search strategy

Six electronic bibliographic databases were searched from January 2000 until September 2019: MEDLINE, Embase, PsycINFO, CINAHL, ASSIA and Sociological abstracts. We selected 2000 as the start date of our searches because it coincides with when the published patient safety research began to increase in volume after the publication of the landmark report To Err is Human: Building a Safer Health System in 1999 [23]. Our search strategy (see Additional file 1) included search combinations of two key blocks of terms: *Patient Safety* and *Marginalised groups*. We used the standardised search strategy for patient safety used in previous patient safety reviews published by our research centre [24]. For the second block of terms, we used a combination of terms derived from two prior reviews on marginalisation (conducted in other topic areas) to represent the concept of marginalisation as well as terms that represent specific groups previously cited as marginalised [7, 25]. We also supplemented these terms with additional terms in order to be as comprehensive as possible. Specifically, the supplementary terms include ‘hard to reach,’ ‘seldom heard’ and ‘vulnerable groups.’

### Eligibility criteria

*Studies were included if they met the following criteria:*

#### Inclusion criteria

- Types of studies: empirical studies and systematic /scoping reviews. Study designs were not restricted and included both quantitative and qualitative studies including case studies;
- Types of participants: Patients who are considered to belong to a marginalised group according to the definition provided above;
- Types of outcomes: data on types of patient safety issues experienced by marginalised people and what factors lead to or were associated with these issues.
- Language: only studies published in the English Language.

#### Exclusion criteria

- Studies concerned with a very specific drug or medical procedure rather than broader categories of patient safety issues;
- Studies concerned with people with a single health condition (unless they also concern a marginalised group);
- Studies that are solely focused on healthcare professionals;
- Studies that are not concerned with health care related safety (e.g. safety in the home, quality of care).

### Study selection

Search results were downloaded first into Endnote and then uploaded and the review process managed via the use of the review software Covidence [26]. All citations deemed relevant after title and abstract screening were retrieved for subsequent review of the full-text article. Studies were assessed for inclusion by two independent reviewers (SCS and GDW) with arbitration by a third reviewer (MP).

### Charting the data

A form was developed by the authors to confirm relevance and to extract key study characteristics such as: 1) publication year, 2) publication type, 3) country, 4) economic level (as classified by the World Bank), 4) study aim, 5) population, 6) key safety outcomes and 7) contributing/associated factors related to the patient safety issues. This form was reviewed by the research team and pretested by all reviewers (SCS, GDW, AP, SG, LR and MP) before implementation. Six independent reviewers were involved in the data extraction. In particular, upon independently reviewing a batch of 20 to 30 articles, the reviewers met to resolve any conflicts and to help ensure consistency between reviewers and with the research question and purpose [27].

### Data synthesis

The data were compiled in a single spreadsheet and imported into Microsoft Excel 2010 (Microsoft Corporation, Redmond, WA) for validation and coding. Studies were then coded and grouped by SCS and GDW (any disagreements were resolved via discussion) according to 1) marginalised group, 2) patient safety issues and 3) contributing or associated factors according to the 7 different factor types from the Framework of Contributory Factors Influencing Clinical Practice within the London Protocol [28] see Table 1. The London Protocol was chosen as it can be applied to all areas of healthcare reflecting the diversity in settings across included studies. Study quality appraisals were not conducted in accordance with standard practice for scoping reviews.

**Table 1:**
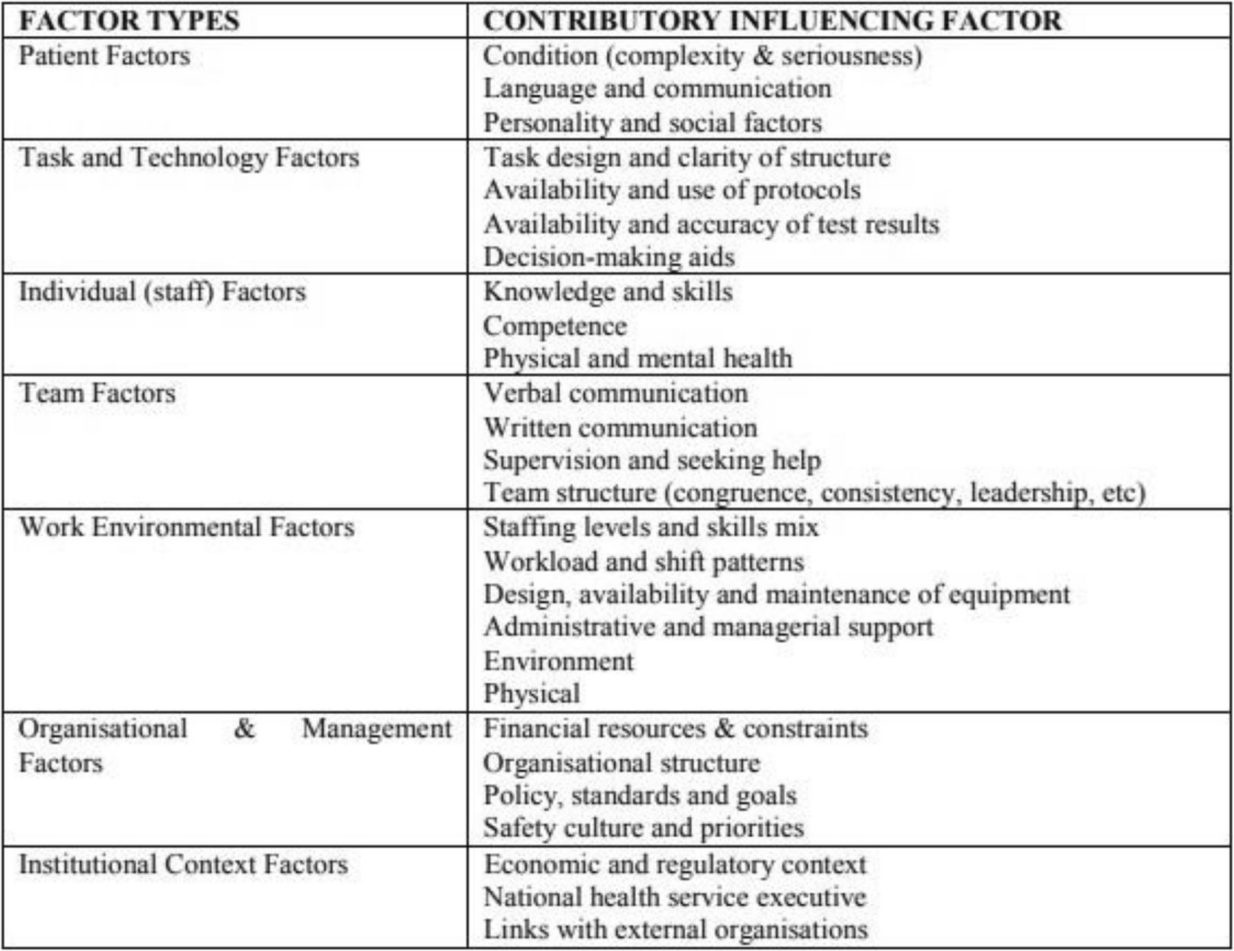
The London Protocol: Framework of Contributory Factors Influencing Clinical Practice

## Results

### Search and selection of studies

**Fig. 1:**
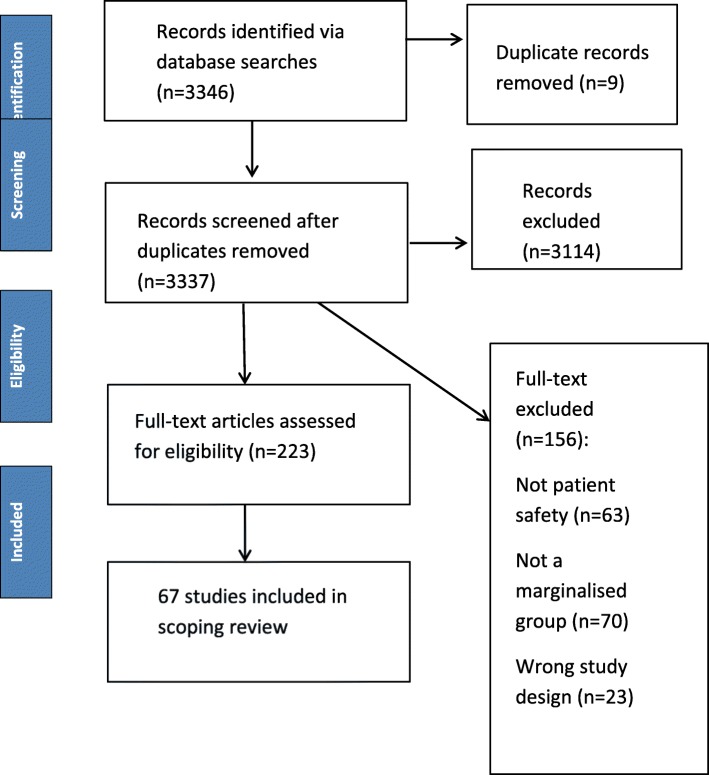
PRISMA flow diagram

The original searches yielded 3346 potentially relevant citations. After completion of deduplication and screening, 67 studies met the eligibility criteria and were included in the review. The flow of articles from identification to final inclusion is presented in Fig. 1.

### Description of general characteristics of included studies

An overview of the included study characteristics is provided in Table 2. All included studies were published between 2002 and July 2019. We identified 8 reviews [29–36] and 59 empirical studies [8, 37–95]. The vast majority of these studies were conducted in high income countries (82%), used a mixture of methods (predominantly quantitative (66%)) and were conducted across multiple settings, with the majority (49%) in secondary care settings. Table 3 (supplementary material) provides details of the individual included studies.

**Table 2:** General Characteristics of Included Studies

General Characteristics of included studies	Number (*n* = 67)	Percentage (%)
Publication Year		
< 2005	3	5
2005–2009	10	15
2010–2014	26	38
> 2015	28	42
Publication type		
Empirical studies	59	89
Reviews	8	11
Economic Level of Study Country		
High	55	82
Upper-Middle	6	10
Lower-Middle	3	5
Low	1	2
Unclear^a^	2	3
Study Setting		
Primary	3	5
Secondary	32	49
Tertiary	2	3
Care home	12	16
Population	4	6
Community	3	3
Multiple	8	11
Not specified/ Unclear	3	5
Study Methods		
Qualitative	13	16
Quantitative	43	67
Mixed (Qualitative and Quantitative)	3	5
Review	8	11

^a^Both studies were reviews which did not extract data to allow the identification of this characteristic

**Table 3:** Description of included studies

Study	Country of study	Economic level	Study Aim	Marginalised Group/Study population(s)	Number of participants/studies (if reviews)	Study setting	Study primary patient safety outcome(s)
Abizanda (2014) [34]	Spain	High	“To analyse the longitudinal association between frailty, disability, multimorbidity or incident disability in a cohort of older adults”	Frail	842	Secondary	Mortality
van Rosse (2016a) [8]	The Netherlands	High	“The following research questions were answered in this study: “1. At which moments during hospitalization do language barriers constitute a risk for patient safety? 2. How are language barriers detected and reported in hospital care? 3. How are language barriers bridged in hospital care? What is the policy and what happens in practice”	Ethnic minority patients	576	Secondary	Communication/ Language barriers
Heyland, 2016 [35]	Canada	High	“The purpose of this paper is to determine the prevalence and nature of errors related to EOL communication and documentation of preferences.”	Frail	808	Secondary	Medical error
van Rosse (2016b) [36]	Netherlands	High	“To analyse the role of these relatives in relation to the safety of patients during hospital care.”	Ethnic minority patients and their relatives (non-western)	576	Secondary	Communication
Storeng (2012) [37]	Burkina Faso	Low	2To challenge “the maternal health literature’s suggestion that women’s health outcomes will be satisfactory if they can access skilled attendance at delivery and emergency obstetric care when pregnancy and delivery complications occur. In this paper, we challenge this assumption through an analysis of the long-term outcomes of ‘Near miss’ obstetric complications.” To “illustrate broader trends that help to explain Burkinabe women’s vulnerability to maternal morbidity and to mortality more generally.”	Women after childbirth in low socioeconomic circumstances	1014	Secondary	Near miss
Beck (2017) [38]	USA	High	“To determine, qualitatively, how the hospitalisation and hospital-to-home transition experiences differed between families of varying SES. Sessions were stratified based on SES, determined by the percentage of individuals living below the federal poverty level in the census tract or neighbourhood in which the family lived.”	Families of low and high socioeconomic status	61	Secondary	Access to care
Ferguson 2015 [39]	USA	High	“The purpose of this research is to identify communication barriers and needs for Deaf and Hard of hearing (HOH) patients when they seek pharmaceutical care, and to better understand the impact of poor communication upon medication adherence and medication errors among this underserved population.”	Deaf/Hard of Hearing (HOH)	20	Community	Communication issues
Cantarero 2014 [40]	Denmark	High	“The aim was twofold: [1] to explore the perceptions, barriers and needs of Arabic-speaking ethnic minorities regarding medicine use, and [2] to use an educationprogram to enhance the knowledge and competencies of the ethnic minorities about the appropriate use of medicines.”	Arabic speaking Ethnic minorities	30	Multiple	Appropriate medicine use
Berry 2017 [41]	USA	High	“This study investigates how family members administered prescription and over-the counter medications to elders with Alzheimer’s”.	Carers	15	N/S	Medication misuse
Groene 2012 [42]	Spain	High	“The objective of our study was to explore the role and engagement of patients in the handover process with a particular focus on these vulnerable patients.”	Vulnerable patients (patients with limited language comprehension or health literacy, or a lack of social resources or support)	12 patients (5 M, 7F) 6 hospital physicians, 5 hospital nurses, 7 primary care physicians, 4 primary care nurses.	Multiple	Discharge
Hole 2015 [43]	Canada	High	Not stated but; “What are community members’ opinions, perspectives, and analysis of the health care system that they interact with, beginning in this instance, at the hospital door?”	Aboriginal people (Syilx people)	28 Aboriginal community members	N/S	Culturally unsafe health care provision
Stenhouse 2013 [44]	UK	High	“To understand the experience of being a patient on an acute psychiatric inpatient ward.”	Psychiatric inpatients	13	Secondary	Inpatient safety
Roost 2009 [87]	Bolivia	Lower-middle	“By means of thematic interviews with women who have experienced severe obstetric complications (Near miss), this study explores the way health care-seeking behaviour is conditioned in an urban Bolivian setting that offers free and easily accessible maternal health care.”	Women from urban (higher SES) and rural (lower SES) who had experienced a Near miss event	30	Secondary care	Near miss
Mohammadi 2017a [45]	Iran	Upper middle	“To explore the experiences of maternal care among Afghan Near miss survivors in Tehran to increase insights into strategies for reducing delays and providing optimal and equitable care for migrants”	Non-indigenous/migrant women and native women	82 women (60 Iranian plus 22 Afghan women)	Secondary	Near miss
Zhi-Han 2017 [46]	Malaysia (Kuala Lumpur)	Upper-middle	“To identify the problems encountered by the visually impaired population when handling their medication.”	Visually impaired	100 (62 blind, 38 visually impaired)	Primary care	Medication safety
Latham 2011 [47]	UK	high	“The purpose of this study was to examine to what extent pharmacy labels follow the existing best practice guidelines of the Design for Patient Safety (DfPS) and The purpose of this experiment was to determine whether labels produced to DfPS guidelines have the potential to be more accessible to patients with impaired vision”	Visually normal patients with simulation to produce visual impairment	20	NS	Medication information
Lahousse 2014 [48]	The Netherlands	High	“The aim was to investigate the prevalence of physically frail elderly in a Dutch population-based cohort study and the impact on adverse health outcomes including all-cause mortality independent of comorbidity.”	Frail	2833	Population	Adverse outcomes
Ekerstad 2017 [49]	Sweden	High	“Early readmissions of frail elderly patients after an episode of hospital care are common and constitute a crucial patient safety outcome. Our purpose was to study the impact of medications on such early re-hospitalizations.”	Frail	408	Secondary	Adverse Drug Reactions
Hastings 2008 [50]	USA	High	“To determine whether frail older adults, based on a deficit accumulation index, are at increased risk of adverse outcomes following discharge from the Emergency Department. Specifically, to examine the association between frailty and [1] any adverse outcome, [2] outpatient ED visits, and [3] more serious events defined as hospitalization, nursing home admission or death.”	Frail	1851	Secondary	Adverse outcomes
Friedman 2008 [51]	USA	High	Previous studies investigating adverse outcomes of hospitalized elders have focused on community-dwelling patients. Given the rapid growth of populations living in other settings, such as assisted living facilities, it is important to understand whether these patients are at higher risk of experiencing specific adverse outcomes during hospitalization, so that interventions can be developed to reduce risk.”	Care Home Residents	212	Care home	Adverse outcomes
Zaal 2013 [52]	Netherlands	High	“ [1] To determine the prevalence of older individuals with an intellectual disability with at least one prescription error and [2] to identify potential risk factors for these prescription errors (age, gender, body mass index (BMI), frailty index, level of intellectual disability and living situation).”	Patients (>/=50y) with Intellectual Disability (IQ </=70), using one or more drugs	600	Primary	Prescribing errors
Shen 2016 [53]	USA	High	“To examine the association between patient race/ethnicity, insurance status, and their interaction with patient safety indicators among hospitalized patients”	Patients of various ethnicities and health insurance status	3,052,268	Secondary	Patient safety indicators (PSIs)
Lin 2011 [54]	Taiwan	High	“To clarify whether intellectual disability (ID) is an independent risk factor for in-hospital major surgeries, and to validate the postoperative adverse outcomes in patients with ID”	Surgical patients with intellectual disability	3983 cases plus ×4 matched controls	Secondary	Adverse outcomes
Marcus 2018 [55]	USA	High	“To examine adverse events and medical errors occurring in VHA hospital psychiatric units”	Psychiatric patients	8005 discharges	Secondary	Patient Safety Indicators (PSIs)
Gaskin 2011 [56]	USA	High	“Employing three years of inpatient discharge data from 11 states and inpatient and patient safety quality indicators from the Agency for Healthcare Research and Quality (AHRQ), this paper explored whether minority (black, Hispanic, and Asian) patients used lower quality hospitals.”	Ethnic minorities	1620	Care home	Patient Safety Indicators (PSIs)
Maly 2011 [57]	USA	High	The purpose of this study was to identify correlates of elapsed time between recognition of breast abnormalities and receipt of definitive diagnosis of breast cancer among low-income women.	Low income women	921	Population	Diagnostic delay
Drumond 2013 [58]	Brazil	Upper middle	“This study aims to evaluate the quality of information about race/colour (black or white) in health information systems and to analyse the causes of infant mortality in the Brazilian List of Avoidable Causes of Death by race/colour in Belo Horizonte in 2001–09.”	Ethnic minority patients	3863	Secondary	Avoidable deaths
Fernandes 2017 [59]	Brazil	Upper middle	“Purpose To evaluate the association between ethnic differences and the occurrence of maternal near miss (MNM) in the Amazon and Northeast regions of Brazil.”	Ethnic Minority mothers and their infants	16,783	Secondary	Near miss
Katzenellenbogen 2013 [60]	Australia	High	“This study aimed to investigate demographic and clinical factors that predict Discharge Against Medical Advice (DAMA) in patients experiencing their first-ever inpatient admission for ischaemic heart disease (IHD). The study focuses particularly on the differences in the risk of DAMA in Aboriginal and non-Aboriginal patients while also investigating other factors in their own right.”	Aboriginal and Non-aboriginal	37,304 people	Secondary	Discharge Against Medical Advice (DAMA)
Haw 2003 [61]	Scotland	High	“To determine the nature, frequency and potential severity of prescribing errors detected by pharmacists working in a psychiatric hospital and to suggest ways that errors might be reduced.”	Psychiatric patients	311 errors	Tertiary care	Prescribing errors
da Costa 2016b [75]	Portugal	High	“To determine the prevalence and nature of Drug-Related Problems (DRPs) in polypharmacy elderly patients residing in nursing homes and to test the acceptability of a pharmacist’s intervention.”	Care Home Residents	126	Care homes	Drug related problems
Garrett 2008 [63]	Australia	High	“While researching the experience of acute patients with limited English, it became evident that some had experienced negative hospital events. This study examined the perception of these negative events, and factors that might prevent or minimise them”	Ethnic minorities	278	Secondary	Communication/ language issues
Khaykin 2010 [64]	United States	High	“The purpose of this study was to determine the association between diagnosis of schizophrenia and adverse events during non-psychiatric hospitalizations.”	Patients with and without schizophrenia	37,362,038 admissions (269,387 non-psychiatric hospitalizations with schizophrenia, and 37,092,651 without)	Secondary	Patient safety indicators (PSIs)
Hubbard 2017 [65]	Australia	High	“We evaluate the predictive validity of the FI-AC in older inpatients. We consider how admission FI-AC score relates to discharge destination and explore its association with other clinically important adverse outcomes.”	Frail	1418 patients	Secondary care	Adverse outcomes
Kandil 2012 [66]	Egypt	Lower-middle	“We designed this study in order to find out the patterns of medication errors and their percentage in an obstetric emergency ward so that we can develop specific strategies to prevent or minimize re-occurrence.”	Women in a low resource setting	10,000 women	Secondary care	Medication error
Bickley 2006 [67]	UK	High income	“To establish the numbers of homeless patients in contact with services who die by suicide; to describe their suicide methods and their social and clinical characteristics including aspects of clinical care”.	Homeless people	131	Multiple	Suicide
Mohammadi 2017b [68]	Iran	Upper middle	“To investigate whether care quality for maternal Near miss (MNM) differed between Iranians and Afghans and identify preventable attributes of MNM”	Non-indigenous/migrant women and native women	76 cases reviewed & 24 MNM mothers interviewed	Secondary	Near Miss
Sarkar 2010 [69]	USA	High	“To examine safety in diabetes patients’ most familiar environment at home, between visits”	Low-income diabetes patients	111	Community	Adverse events
Stadjuhar 2019 [91]	Canada	High	“The purpose of this study was to identify barriers to accessing care among structurally vulnerable people at EOL.	Vulnerable end of life patients	25 people experiencing structural vulnerability, 25 support persons, and 69 formal service providers	Community (End of life care)	Access
Gamlin 2018 [94]	Mexico	Upper Middle	“This study aimed to explore the structural determinants of maternal and perinatal health in a Wixárika community located in Northwestern Mexico.”	Pregnant indigenous women	62 pregnant women	Community	Mortality
Komiya 2018 [92]	Japan	High	“we investigated the demographic and clinical factors associated with polypharmacy in elderly care home patients, which we expected to be different from those of outpatients, because elderly care home patients are more likely to be frail than outpatients and are not prescribed medications by multiple clinical departments”	Care Home Residents	153 care home residents	Care Homes	Polypharmacy
Katikireddi 2018 [93]	UK	High	Not specified	Ethnicity	4·61 million Scottish people	Population	Adverse Outcomes and Mortality
Funk 2018 [95]	USA	High	“This study was conducted to assess the hospital experience of older adults with hearing impairment, and to use the findings in formulating suggestions for improving nursing care.”	Deaf/Hard of Hearing (HOH)	8 older (> 65 yr) adults with self-reported hearing impairment	Secondary	Communication issues
Bennett 2014 [70]	Australia	High i	“In older robust and frail patients admitted to hospital after a fall, we investigated the prevalence and clinical impact of fall-risk-increasing drugs (FRIDs), total number of medications, and drug–drug interactions (DDIs)”.	Frail	204	Tertiary care	Adverse outcomes
deBruijne 2013 [71]	Netheralnds	High	“We explored variations in indicators of quality of hospital care by ethnicity in the Netherlands.”	Ethnic minority patients	433,501 patients in 139 hospital locations	Secondary	Adverse outcomes
Poudel 2016 [72]	Australia	High income	“We aimed to determine the prevalence of polypharmacy and its association with adverse outcomes in hospitalized older patients and to assess the additional role of frailty”	Frail	1418	Secondary	Adverse Outcomes
Reime 2012 [73]	Germany	High	“To examine the association between region of origin and severe illness bringing a mother close to death (Near miss).” “We examined the association between maternal regional origin and four indicators of maternal Near miss, namely hysterectomy, haemorrhage, sepsis and eclampsia, first to describe the association between maternal regional origin and near miss complications and second to test whether higher risks among women with migrant background can be explained by the women’s socioeconomic characteristics, health-related behaviour and pre-existing maternal conditions”	Migrant mothers	441,199	Secondary	Near miss
Bronskill 2012 [74]	Canada	High income	“The use of multiple, concurrent drug therapies often referred to as polypharmacy, is a concern in long term care (LTC) setting, where frail older adults are particularly at risk of dose adverse events. We quantified the scope of this practice by exploring variation in the use of nine or more drug therapies across LTC homes.	Care home residents	64,394 residents in 589 care homes.	Care homes	Polypharmacy
da Costa 2016a [62]	Portugal	High	“Objective This study aimed to determine the prevalence of Potentially Inappropriate medications (PIMs) and potential prescribing omissions (PPOs) in a sample of Portuguese nursing homes residents.”	Care home residents	161	Care home	Potentially Inappropriate Medications
Desai 2013 [76]	UK	High	“The objectives of this study were [1] to characterize nursing home anticoagulant medication errors, [2] to study their causes and outcomes, and [3] to evaluate their association with patient harm.”	Care home residents	32,176 individual medication error incidents	Care homes	Medication errors
DeVylder 2015 [77]	USA	High	“To examine the association between 12-month suicidality and 12-month psychotic experiences and to test the hypotheses that psychotic experiences are associated with increased prevalence of suicidal ideation and suicide attempts during the concurrent period and with greater severity of suicidal behaviour.”	Psychiatric patients	11,716	Population	Twelve-month suicidal ideation and suicide attempts
Hoffman 2003 [78]	USA	High income	This project was undertaken to reduce the inappropriate use of bed rails in a long-term care facility without increasing the risk of injuries caused by falls	Care home residents	180 beds	Care home	Adverse outcomes
Cromwell 2005 [79]	USA	High	The objective of this study was to explain race/ethnic disparities in hospitalizations, utilization of high-technology diagnostic and revascularization services, and mortality of elderly ischemic heart disease (IHD) patients.	Ethnic minority patients	700,000	Secondary	Access to care
Dent 2014 [80]	Australia	High	“The aims of this study were to: i) investigate the association between psychosocial factors and frailty, and ii) to establish whether psychosocial factors impact on the association between frailty and adverse outcomes.”	Frail	172	Secondary	Adverse outcome
van Rosse 2014 [81]	Netherlands	High	“1) Assess the risk of AEs for hospitalised patients of non-Western ethnic origin in comparison to ethnic Dutch patients; 2) analyse what patient-related determinants affect the risk of AEs; 3) explore the mechanisms of patient-provider interactions that may increase the risk of AEs; 4) explore possible strategies to prevent inequalities in patient safety”	Dutch and Ethnic minority patients	1339 (763 Dutch patients and 576 ethnic minority patients)	Secondary	Adverse events
Briesacher 2005 [82]	USA	High income	“To test whether nationally required drug use reviews reduce exposure to inappropriate medications in nursing homes”.	Care home residents	Nationally representative population sample of 8 million nursing home (NH) residents (unweighted n52,242) and a comparative group of 2 million assisted living facility (ALF) residents (unweighted n5664).	Care homes	Adverse drug reactions
Boockavar 2004 [83]	USA	High income	“To identify medication changes during transfer between hospital and nursing home and adverse drug events (ADEs) caused by these changes”.	Care home residents	87 residents	Multiple	Adverse drug Events
Adisasmita 2015 [84]	Indonesia	Lower-middle	“To evaluate the associations between maternal demographic and health care related characteristics and obstetric diagnoses with mode of delivery and Near miss and death and To predict outcomes based on socio-economic status, personal barriers, access barriers to care and referral patterns”	Low and high income pregnant women	1358 retrospective (no socio-economic data) + 1240 prospective (Interview data only on 56.8%).	Secondary	Near miss
Castle and Engberg 2007 [85]	USA	High income	“We examine deficiency citations for medication use, with an emphasis on psychoactive drug use”.	Care home residents	16,533 facilities	Care homes	Inappropriate use of medication
Pepper 2007 [27]	NS	Unclear as countries not stated	“The purpose of this research synthesis was to examine the published literature to develop estimates of the incidence, severity and costs of medication errors in nursing homes and other institutional long-term care settings.”	Care home residents	65	Care homes	Medication errors
Almeida 2013 [28]	Studies from Europe, USA, Canada, Australia	high (all included)	“To review the existing scientific evidence on the access, use and quality of healthcare in migrant populations during pregnancy and postpartum period, with particular emphasis on how this interferes with health indicators and outcomes”	Migrant women	30 studies	Population	Access
Khanassov 2016 [29]	USA, Canada, UK, New Zealand, Australia, Italy	High	“The purpose of this scoping review was to describe the nature and breadth of published research studies in peer reviewed academic journals on organisational interventions improving access to primary care services for vulnerable populations, and reducing consequences of poor access in these populations.”	Vulnerable populations	39 studies	Primary care	Access
Alhomoud 2013 [30]	UK	High	“To review and establish types and possible causes of medicine related problems experienced by ethnic minorities in the UK”	Ethnic minority patients	15 studies	Multiple	Medicine related problems
Castro 2015 [31]	Latin America	Upper middle	“To identify and understand the barriers to equitable care within health care settings that women of ethnic minorities encounter in Latin America and to examine possible strategies for mitigating the issues.”	Women of ethnic minorities	60 studies	Multiple	Communication
Corsonello 2009 [32]	Italy	High	“Therefore, we will review the evidence pertaining to the application of Beers’ criteria in elderly hospitalized patients, while focusing on Italian studies that have investigated the role of PIMs as potential predictors of negative hospital outcomes. In addition, we will also review the available evidence regarding new European criteria on identifying PIMs, because clinical application in elderly hospitalized Europeans is still under investigation.”	Frail	5 studies	Secondary	Potentially inappropriate medication
Hemsley 2014 [33]	N/S	N/S	“The aims of the present review were to map the evidence on communication in hospital for patients with severe communication disabilities; to synthesize the findings of original relevant research in order to propose an evidence-based set of core strategies suggested to improve communication, and to propose a translational research agenda to improve communication in hospital. This includes raising the awareness in all stakeholders for the need for proper evaluation of any suggested strategies to improve hospital communication for this population”	Patients with severe communication disabilities	18 studies	Multiple	Communication/ language issues
Hoffmann 2019	Germany	High	“our aim was to give an overview on the existing literature on a) the occurrence of hospitalizations at the end of life in NHR with dementia and b) to compare these figures to NHR without dementia in the subset of studies reporting both groups.”	End of life nursing home residents with dementia	13 studies	Multiple	Adverse Outcomes (hospitalisation)

### Description of marginalised groups

We identified 13 different marginalised groups within the identified literature (see Table 4). Over two thirds of studies (69%) concerned just four marginalised groups. The largest of these (constituting over a quarter of studies (26%)) focused on ethnic minority groups [8, 32, 33, 39, 43, 46, 56, 59, 61–63, 66, 74, 82, 84, 93, 94], those residing in care homes (18%) [29, 32, 36, 54, 65, 77–79, 81, 85, 86, 88], followed by frail elderly populations (15%) [34, 37, 38, 51–53, 68, 73, 75, 83, 91, 92, 95], and individuals of low socio-economic status (10%) [40, 41, 60, 69, 72, 87, 90].

**Table 4:** Type and frequency of marginalised groups and patient safety issues identified in included studies

Marginalised Group	Frequency (%)
Ethnic minorities^a^	17 (25)
Frail elderly	10 (15)
Care home residents	12 (18)
Low socio-economic status	7 (10)
Psychiatric patients	5 (7)
Migrants	4 (6)
Vulnerable patients	3 (4)
Visually Impaired	2 (3)
Intellectually Disabled	2 (3)
Carers	1(2)
Homeless	1 (2)
Deaf/Hard of Hearing	2 (3)
Communication Impaired	1 (2)
Patient Safety Issue^b^	Frequency (%)
Medication safety issues	18 (26)
Adverse outcomes	15 (24)
Near Miss	7 (10)
Language/communication issues	6 (9)
Access to care	5 (7)
Patient safety Incidents/Indicators	4 (6)
Mortality (including Suicide & avoidable death)	6 (9)
Discharge Safety	2 (3)
Medical Error	1 (2)
Culturally unsafe healthcare	1 (2)
diagnostic delay	1 (2)
Inpatient safety	1 (2)

^a^Includes aboriginal/indigenous populations

^b^ The denominator is 68 here as one study had two different outcomes (mortality and adverse outcomes)

### Description of patient safety issues

We identified 12 separate patient safety issues (see Table 4) within the included studies. Over half of the studies concerned three major patient safety topics. The largest of these, (constituting just over a quarter of the studies (28%)) focused on varying aspects of medication safety [29, 32, 34, 43, 44, 49, 50, 55, 64, 65, 69, 77–79, 85, 86, 88, 92], followed by adverse outcomes (e.g. increased risk of hospital re-admission) (22% of all studies) [36, 51–54, 57, 68, 72–75, 81, 83, 84, 93] and near miss in maternal care (10%) [40, 48, 62, 71, 76, 87, 90, 94].

### Overview of marginalised groups and patient safety issues

**Fig. 2:**
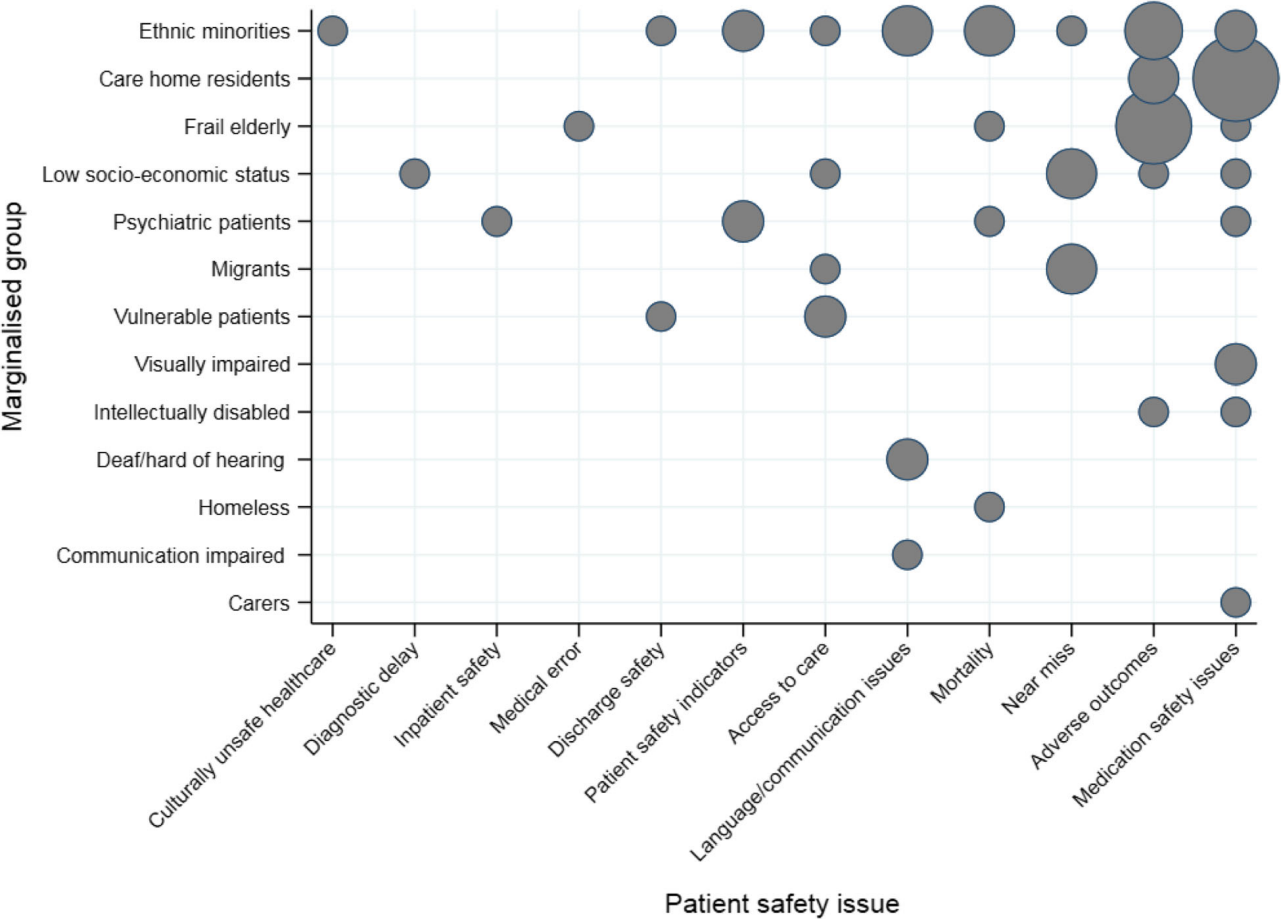
Bubble plot of the distribution of identified patient safety issues and marginalised groups in included studies

Figure 2 represents the distribution of patient safety issues and marginalised groups identified across included studies. Most patient safety issues (9/12) were repeatedly reported across more than one study except for four unique issues (culturally unsafe healthcare^1^ [46], diagnostic delay [60], inpatient safety [47] and medical error [38]). Similarly, most marginalised groups (9/13) were studied in more than one study. The largest proportion of studies were in two areas, 1) medication safety issues in care home residents [29, 54, 65, 77–79, 81, 85, 86, 88, 92] and 2) studies of adverse outcomes in frail elderly populations [51–53, 68, 73, 75, 83].

### Description of contributory/associated factors

In total, 157 factors,^2^ mapped to one of 7 different factor types (from the London Protocol), contributed to or were associated with patient safety issues (see Table 5). In the vast majority of studies (52 or 78%) the identified feature(s) of marginalisation (e.g. a patient factor such as frailty) led to patients in that group experiencing negative implications for their patient safety thereby leading authors to conclude that the characteristic itself was a contributing/associated factor to the patient safety issue of interest. Four studies reported no discernible/neutral effect [36, 59, 84, 92], two indicated a positive effect on patient safety [55, 88] and one mixed effects as two outcomes were measured and had different directions [93]. In 7 studies, no factors were identified [31, 34, 50, 58, 65, 78, 81] and in two, it was unclear [44]. Most studies reporting factors, discussed multiple individual factors (range = 1–7, average = 2.3) across multiple domains (range = 1–4, average = 2.0). The single largest domain concerned patient factors with 95 counts followed by individual staff factors (*n* = 27) and institutional context (*n* = 16). A brief summary of examples in each factor type is presented beneath and ordered by frequency from highest to lowest count.

**Table 5:** Contributory and/or associated factors to patient safety issue occurrence across included studies

Study (first author, year)	Institutional Context Factors [ICF]	Organisational & Management Factors [OMF]	Work Environmental Factors [WEF]	Task and Technology Factors [TTF]	Individual (staff) Factors [ISF]	Team Factors [TF]	Patient factors [P]	Factor examples	Implied direction of factor(s) effect for patient safety
Storeng (2012) [37]	1							[ICF] Payments for care	Negative
Maly (2011) [57]					1		3	[P]: 1) Race 2) patient self-efficacy and3) cultural beliefs affecting care. [ISF]: clinical breast examination had longer delay than by mammogram.	Negative
Abizanda (2014) [34]		1					3	[P]: 1) Frailty, 2) Institutionalisation and 3) disability	Negative
Beck (2017) [38]	1							[ICF] Payments for care	Negative
Cromwell (2005) [79]							1	[P] Race	Negative
da Costa (2016a) [62]								None measured	Not applicable
da Costa (2016b) [75]								None measured	Not applicable
DeVylder (2015) [77]							2	[P]:1) Psychosis and 2)suicidality	Negative
deBruijne (2013) [71]	1						1	[P]:Ethnicity and Payments for care [ICF]	Negative
Dent (2014) [80]							1	[P]:Frailty	Negative
Desai (2013) [76]							2	[P]:Age, and mental capacity (cognitive ability)	Negative
Ekerstad (2017) [49]	1						2	[ICF:]Under-use evidence-based drug treatment and [P]: 1) heart failure and 2) anaemia were predictors for readmission	Negative
Friedman (2008) [51]							6	[P]: 1) Worsening function, 2) delirium, 3) depression, 4) falls, 5) pressure sores, and 6) admission from a nursing home.	Negative
Garrett (2008) [63]			2				1	[WEF] staff workload/pressures and staff neglect, [P] communication/language	Negative
Gaskin (2011) [56]							1	[P] No associations for ethnicity	Neutral
Groene (2012) [42]	1	1						[OMF]: No commonly accepted standard operating procedures for the exchange of information between secondary care and primary care. Communication and role of patient within discharge variable and unclear [ICF]	Negative
Hastings (2008) [50]							1	[P] Frailty	Negative
Haw 2003 [61]					2			[ISF] Decision-making errors and Errors in prescription writing	Negative
Heyland, 2016 [35]							2	[P] Frailty and social support	Negative
Hole 2015 [43]	1						2	[P] perceived discrimination and Interpersonal experiences of marginalization (e.g. not being listened to/believed judged in a negative light)) [P]. [ICF] Structural factors.	Negative
Hubbard 2017 [65]							1	[P] Frailty	Negative
Katzenellenbogen (2013) [60]							3	[P]: emergency admission, alcohol admission with or without mental health-related admission history and Aboriginality	Negative
Khaykin (2010) [64]						1	1	[P] Schizophrenia and [TF] effective communication among healthcare providers and between health care providers and this vulnerable patient population.	Negative
Lahousse (2014) [48]							1	Access to care - hospital care unaffordable [ICF], poor women-professional communication [ISF], obstetric professionals busy and lack of time [WEF]; mothers felt clinical team unqualified to diagnose illness [P]; low education levels /illiteracy [P]	Negative
Latham (2011) [47]								None measured	Not applicable
Lin (2011) [54]							1	[P] Intellectual Disability patients - Complications correlated with ID severity, especially in septicaemia.	Negative
Marcus (2018) [55]								None stated - measures of events, preventability and harm but not cause.	Not applicable
Reime (2012) [73]							1	[P] ethnicity (Women from the Middle East, Asia and Africa/Latin America vs. women from Germany). These differences were not explained by the sociodemographic, behavioural or health-related factors.	Negative
Sarkar (2010) [69]	1				2		2	[ICF] Systems issues, patient physician communication problems [P] + [ISF], and clinician [ISF] and patient actions-[P]	Negative
Shen (2016) [53]							1	[P] Poverty	Negative
Stenhouse (2013) [44]		1						[OMF] No perceived polices for safeguarding	Negative
van Rosse (2016a) [8]					2		1	[ISF]1) daily clinician practices e.g. ‘drop-out’ of protocolised name and/or date-of-birth checks not done during critical care moments due to language barriers and 2) lack of use of professional interpreters despite 3/4 hospitals having an explicit policy to encourage use (policies not enacted). Language barriers [P]	Negative
van Rosse (2016b) [36]					2		1	[P] language and communication issues due to role of relatives	Unclear
Van Rosse (2014) [81]							1	There was no significant difference in the incidence of AEs in Dutch patients and in ethnic minority patients [P].	Neutral
Zaal (2013) [52]		1			1		1	[ISF] physicians may prescribe drugs more carefully to individuals with a more severe ID, resulting in fewer errors. [P] Individuals with a more severe ID are being treated in centralized settings [P]. [OMF] Centralised settings employ specialized physicians for people with intellectual disabilities more often.	Positive
Bennett (2014) [70]							1	[P] Frailty	Negative
Berry (2017) [41]							3	[P] 1) Cognitive decline (Alzheimer’s), 2) social support (as carers taking over medication management) and 3) elder resistance to medication-taking.	Unclear
Bickley (2006) [67]			2		1	1	1	lack of supervision [WEF], poor patient compliance with medication [P], knowledge of staff [ISF], staffing levels [WEF] and poor communication [TF].	Negative
Boockavar (2004) [83]	1							Transitions/discharge related medication issues (between hospitals and nursing homes) [ICF]	Negative
Briesacher (2005) [82]	1	1						[ICF] National policy changes designed to affect the use of potentially inappropriate medications and implementation practices of care homes [OMF] led to variation in prescribing of potentially inappropriate medications.	Negative
Bronskill (2012) [74]		1						[OMF] variation in polypharmacy rates across care homes	Negative
Cantarero (2014) [40]							4	[P] Multiple perceptions of medicines and medicine-related problems: 1) not taking meds from Danish doctors, inherited incorrect information from their parents, 2) perceived differences in treatment from doctors due to foreign status, 3) impossible to understand the instructions and recommendations of their doctor in Danish and 4) specific needs concerning appropriate medicine use and information.	Negative
Castle and Engberg (2007) [85]		1						[OMF] Size of the nursing home and [ICF] Medicaid reimbursement rates.	Positive
Ferguson (2015) [39]					1		2	[P] 1) unable to communicate due to deafness/Hard of hearing (HOH) and 2) experiencing an adverse event due to deafness/HOH and [ISF] perceived lack of sensitivity by pharmacists	Negative
Hoffman (2003) [78]								None stated	Not applicable
Poudel (2016) [72]								[P] Frailty	Negative
Adisasmita (2015) [84]		1			1		2	[P] 1) Poverty and 2) delivery outside the hospital are significant risk factors associated with near miss. Hospital/staff practices [ISF] and [OMF] response time.	Negative
Kandil (2012) [66]					4			[ISF] Administration errors were either due to: wrong 1) rate 2) dose, 3) route or 4) time of administration of the drug.	Negative
Roost (2009) [87]							3	[P] 1) Strategies shaped by family traditions and composed experiences 2) The perception of not belonging (lack of knowledge, fears of hospital); 3) Mistreatment and distrust.	Negative
Drumond 2013 [58]							2	[P]: Ethnicity and socioeconomic status.	Negative
Fernandes 2017 [59]	1							[ICF] Inadequate healthcare access	Negative
Mohammadi 2017a [45]	1		1		1		4	[ICF] Access to care - hospital care unaffordable, poor women-professional communication [ISF], obstetric professionals busy and lack of time [WEF]; mothers felt clinical team unqualified to diagnose illness [P]; low education levels /illiteracy [P] cited as an issue. Lack of understanding caused women to not question health professionals [P]. discrimination - voice not being heard particularly by midwives and feeling as though treated differently [P].	Negative
Mohammadi (2017b) [68]							4	Illiteracy [P] and having only primary education [P], low income status [P] and being Afghan [P]	Negative
Zhi-Han (2017) [46]							1	[P] vision problems (inability to read the prescription labels)	Negative
Corsonello (2009) [32]								none stated	Not applicable
Khanassov (2016) [29]								None stated	Not applicable
Pepper 2007 [27]							5	[P]: 1) female sex, 2) caucasian,3) great number of medication prescriptions, 4) age less than 85 and 5) not having cognitive impairment.	Negative
Castro 2015 [31]	1				1			[ISF] Poor communication derived from healthcare professionals not communicating in indigenous languages and resulting in poor quality access to healthcare [ICF].	Negative
Hemsley 2014 [33]	1		1	2	2			(a) services, systems, and policies needed that support improved communication [ICF], (b) enough time to communication [WEF], (c) ensure adequate access to communication tools (nurse call systems and communication aids [TTF], (d) access personally held written health information [TTF], (e) collaborate effectively with carers, spouses, and parents,[ISF] and (f) increase the communicative competence of hospital staff [ISF].	Negative
Alhomoud 2013 [30]					1		3	[P]:1) In ethnic minority groups differing cultural perceptions or beliefs about health, illness, prescribed treatment and medical care impact on the use of medicines. 2) Ethnic minority groups have different experiences, needs, values and expectations of illness, prescribed treatment and medical care 3) Language and communication barriers have been identified as a possible contributory factor to Medicine Related Problems.[ISF]: inability to communicate in what is not the ethnic minorities’ mother tongue may lead to discrimination	Negative
Almeida 2013 [28]	1				2		2	[ICF]: reduced access to health facilities. [ISF]:1) poor communication between providers and patients and 2) less follow-up. [P] 1) higher health risk profile in immigrants and 2) high likelihood of comorbidities.	Negative
Hoffmann 2019 [36]							3	[P]: 1) Age, 2) gender and 3) condition (dementia vs. non-dementia)	Unclear
Stajduhar 2019 [91]	2	1			1		1	[ICF]: 1) social disadvantages and oppressions and 2) The cracks of a ‘silo-ed’ care system. [P]: The normalization of dying (form of fatalism). [ISF]:The problem of identification [OMF] Professional risk and safety management	Negative
Komiya 2018 [92]							7	[P]: 1) lower care need level, 2) higher Barthel Index (BI), 3) higher Mini-Nutritional Self-Assessment Short Form (MNA-SF), 4) lower Charlson Comorbidity Index (CCI), 5) the presence of Potentially Inappropriate Medicines (PIM), 6) the presence of pollakisuria, 7) presence of insomnia	Unclear
Katikireddi 2018 [93]							1	[P]: Ethnicity	Mixed effects
Gamlin 2018 [94]			1		2		1	[WEF]: 1) the structure of service provision, in which providers have several contiguous days off, [ISF] 1) poor patient-provider dynamic and discriminatory practices and 2) sometimes non-consensual imposition of biomedical practices. [P] men have important roles to play supporting their partners during labour and birth.	Negative
Funk 2018 [95]							3	[P]: 1) Health care communication difficulties due to patient non-disclosure of condition, 2) passivity and vulnerability, and 3) frustration with family	Negative

#### Patient factors (*n* = 95)

This was the largest factor type, with 61% of all individual factors being identified as belonging to this category. We classified any contributing or associated factors that were either intrinsic to the patient or as a result of their social/economic/cultural characteristics as belonging to this factor type. There was wide variation in the types of examples, but a patients’ race/ethnicity, their condition (mental and/or physical e.g. frailty, disability), issues in communication capabilities (language, disability or literacy) and help-seeking behaviour (e.g. route of admission, cultural beliefs, how they perceived themselves to be treated by clinical staff) were the largest sub-categories within this factor type.

#### Individual (staff) factors (*n* = 27)

Communication skills (e.g. perceived behaviour/manner towards patients) issues as well as knowledge/cognition based errors (e.g. errors in prescribing) were most commonly identified amongst coded examples. A lack of policy adherence/enactment by clinicians was also identified. However, an example of how this factor can positively impact on patient safety was seen in one study which hypothesised the outcome to be due to recognition of patient vulnerability (arising from their intellectual disability) resulting in more considered/careful behaviour by clinicians.

#### Institutional context factors (*n* = 16)

Access to care was the largest example of this factor, particularly access being moderated by the requirement for patients to make (co-)payments in order to access care. Policies in terms of a lack of, or lack of enactment as well as issues in transitions of care (e.g. lack of consideration and responsiveness to patient factors) were also identified as leading to patient safety issues occurring.

#### Organisational & Management Factors (*n* = 8)

Organisational policies availability and their variation in implementation was the primary example of this factor. Organisational size, specialisation i.e. staff and patient type within the organisation and responsiveness were also identified as impacting on patient safety.

#### Work environmental factors (*n* = 7)

Staff workload, shortages and time pressures led to patients’ perceptions of staff ‘busyness’ and in one case, perceived patient neglect formed the coded examples in this factor type.

#### Task and technology factors (*n* = 2)

Only two occurrences of this factor were identified (the availability of communication tools and personally held written health information) and both arose from the same study concerning patients with communication impairment/disability.

#### Team factors (*n* = 2)

Only two examples of this factor were identified, across two separate studies and both concerned team communication.

## Discussion

This scoping review brings together the published academic literature regarding patient safety in marginalised groups and included 67 studies in total. Most studies were from high-income countries and were quantitative (observational) in nature, designed to ascertain whether or not there was a discernible impact on patient safety as a result of the marginalised groups characteristics(s) investigated. Results revealed that in most cases, multiple contributing factors and factor types linked to marginalisation, appeared to lead to negative implications for patient safety. Medication related safety issues and studies around ethnicity constituted the two largest areas with existing evidence. This coalescence however also leaves many gaps in knowledge in the literature allowing for new research agendas to be clearly identified. What is clear, is that there is a relative paucity of patient safety research conducted with respect to marginalised groups in general and that this aligns with a recent priority setting exercise that highlighted vulnerable patients as the top research priority for patient safety research in primary care [96].

Common to studies showing a negative impact on patient safety, was the finding that the studied attributes from the particular marginalised group of interest and their interaction with the health system, created spaces or ‘safety vulnerabilities’ for patient safety issues to occur (or to be more likely to occur). In mapping the studies reviewed to categories according to the London Protocol Framework, the results of this review point to patient factors being the primary area as to where these vulnerabilities occur. However, many of these patient factors are not transmutable and are necessarily tied to social and organisational context [97], therefore an attempts to improve patient safety for people from marginalised groups requires the system and those working within it to respond and change appropriately. On the basis of the current evidence identified in this review systems, organisations, and those working within it, were for the most part seemingly unable to compensate for or respond adequately to these patient factors and our review highlights that the reasons for this (e.g. work-environmental factors, team factors) have not been well studied.

Access to high quality, safe health care is a fundamental indicator societal and health equity. The findings of this review highlight the need for high quality research to understand the patient, health provider and systemic factors which explain the present inability of health care organisations to provide high and equitable standards of care and safety to marginalised patients. Given that most incident reporting systems are limited in scope [98], explicitly listing marginalised patient groups at high risk for patient safety incidents requires immediate attention by policymakers and practitioners.

An important research implication is the need to acquire a deeper understanding of the underlying vulnerabilities of patient safety in marginalised groups of patients and design improvement strategies. Such understanding and improvements will require researchers to study and address the multi-factorial nature of patient safety issues and their occurrences drawing from a range of disciplines in order to address the multiple factors and issues identified ranging from the micro-level patient-provider interaction to specific and innovative service design to address macro-level issues such as the reduced access to care experienced by people from marginalised groups. A number of possible avenues could be productive. Firstly, existing theoretical frameworks can support a critical consideration of the relationships between patient factors, clinical interactions and wider organisational context of systems within patient safety research. For example, the social model of disability makes an important distinction between bodily impairment and disability and associated disadvantage created by environmental and social exclusion [99]. Secondly,the distinction between medical and social models also resonates with previous qualitative research on patient safety highlighting the tendency of patients to highlight the importance of psychosocial aspects of safety such as trust, communication and continuity [100]. Such issues are likely to be even greater concerns for groups where there is little current evidence, such as those with mental health problems, communication and cognitive impairments, or in specific contexts such as homelessness. In addition, research focusing on such groups entails consideration of *intersectionality* where multiple social markers (e.g. age, gender, ethnicity, socio-economic status) may synergistically influence the degree to which people are marginalised, vulnerable, excluded or disadvantaged within care systems [101]. Exploration of these issues (quantitatively and qualitatively) will promote further understanding of the overlaps and distinctions regarding marginalisation and vulnerability, as well as an understanding of amenable contributors to patient safety.

The identification and understanding of amenable factors for patient safety provides a crucial base for generating solutions and draws attention to additional avenue for further research focused on marginalised groups and patient safety: the co-design and evaluation of appropriate interventions to improve the quality and safety of care. Whilst there has been a growing acknowledgement on the need for patient and public involvement and engagement to achieve such improvements there is limited evidence of such work, even in relation to black and minority ethnic groups representing the largest marginalised group focused on in the literature reviewed here [102, 103]. Furthermore, the drive for increasing digitisation within care services in many high income countries [104, 105] can potentially increase any existing inequalities [106] and indeed create new and as yet unknown patient safety issues for marginalised people(s) [107]. Conversely, there are potentially opportunities for digital technology to reduce inequalities e.g. service gap provision. What is clear however, is that the development of any technology designed to ameliorate patient safety issues for marginalised people(s), will have to first understand the specific issues as a basis for co-design. This entails a focus on multiple dimensions of experience as discussed above; for example physical impairment as well as the material and interactional contexts where technologies are deployed [108].

New research to improve knowledge and understanding of patient safety risks for marginalised groups would also allow policymakers access to information as to where patient safety vulnerabilities are occurring and enable more effective planning and system responsiveness as well as evidence-based policies of inclusion, particularly those that recognize inequities in resources [109]. What is clear from this review, is that the field of patient safety research for marginalised groups has much scope for research, with many areas of patient safety and groups being under-researched.

### Strengths and limitations

This is the first attempt to identify and analyse the academic literature for patient safety within marginalised groups. The study provides a clear platform for further research by highlighting where the gaps in literature are. We conducted systematic searches and double screened all studies. Identifying studies and key words for marginalised groups however was challenging. Thus, there is a possibility that some relevant studies were not included despite thorough attempts to do so. In addition, our focus on studies of marginalised groups meant that we excluded studies where health professionals were the focus and their views may have been different to those of the patients within marginalised groups. Only including studies in the English Language will have also affected the range of possible included studies and consequently meant that majority of studies were from high income countries. Furthermore, defining marginalisation is difficult and often overlaps with other concepts such as vulnerability. We have tried to be inclusive and used search terms from prior published reviews (and appropriate inclusion and exclusion criteria) and we established inter-rater agreement whilst determining the eligibility of the studies but admittedly operationalising marginalisation involves some degree of subjectivity. Finally, although we found that in the majority of cases, the features of marginalisation in the included studies appeared to lead to negative implications for patient safety for marginalised groups, we cannot say what the strength of this relationship is as scoping reviews do not aim to produce a critically appraised and synthesised result.

## Conclusions

Our review identified a range of patient safety issues for people in marginalised groups, whether these groups are defined by social, economic, demographic or by other means of stratification. The findings indicate the need for further research to understand the intersectional nature of marginalisation and the multi-dimensional nature of patient safety issues, for groups that have been under-researched, including those with mental health problems, communication and cognitive impairments. Understanding which groups in particular are most likely to experience safety issues, what these issues are and why they occur in turn provides a basis for working collaboratively to co-design training, services and/or interventions designed to remove or at the very least minimise these increased risks.

## Supplementary information

**Additional file 1.** Search Strategy

## Data Availability

All data generated or analysed during this study are included in this published article [and its supplementary information files].

## Abbreviations

NHS: National health service
OECD: Organisation for economic co-operation and development
PPI: Patient and public involvement

## Footnotes

1 This study defined culturally unsafe practice as any actions that diminish, demean, or disempower the cultural identity and well-being of an individual and applied this concept to health care.

2 Within studies, multiple factors were counted individually e.g. if ethnicity and the person’s condition where both identified, then these would be counted separately within the patient domain. Additionally, where studies indicated multiple instances of the same individual factor, these were also counted separately.
