# Supplementary material for: Patient safety in marginalised groups: a narrative scoping review
Source: Int J Equity Health. 2020 Feb 12;19:26. doi: 10.1186/s12939-019-1103-2 (PMC7014732; doi:10.1186/s12939-019-1103-2)
Supplement: Supplementary file 1 — Additional file 1. Search Strategy [file 12939_2019_1103_MOESM1_ESM.docx]

| \| **#** \| **MEDLINE Searches** \| \| **Results** \| \| --- \| --- \| --- \| --- \| \| 1 \| exp malpractice/ \| \| 30781 \| \| 2 \| patient safety.tw. \| \| 19034 \| \| 3 \| safety culture.tw. \| \| 1198 \| \| 4 \| (safe$ adj2 (practice$ or manage$)).tw. \| \| 8869 \| \| 5 \| iatrogenic disease$.tw. \| \| 662 \| \| 6 \| malpractice$.tw. \| \| 8759 \| \| 7 \| (patient adj2 harm$).tw. \| \| 1403 \| \| 8 \| human error$.tw. \| \| 1585 \| \| 9 \| ((service$ or system$ or communication$ or organisation$ or organization$) adj1 (weak$ or fail$)).tw. \| \| 4130 \| \| 10 \| (latent adj1 (threat$ or cause$ or fail$)).tw. \| \| 182 \| \| 11 \| ((medica$ or diagnostic or therapeautic or administration or dispensing or prescri$) adj1 (error$ or mistake$ or fault$)).tw. \| \| 11553 \| \| 12 \| near miss$.tw. \| \| 1598 \| \| 13 \| never event*.mp. or adverse event*.tw. [mp=title, abstract, original title, name of substance word, subject heading word, floating sub-heading word, keyword heading word, protocol supplementary concept word, rare disease supplementary concept word, unique identifier, synonyms] \| \| 107376 \| \| 14 \| untoward incident*.tw. \| \| 49 \| \| 15 \| serious incident*.tw. \| \| 150 \| \| 16 \| adverse event*.tw. \| \| 107163 \| \| 17 \| Medical Errors/ \| \| 15244 \| \| 18 \| negligence.mp. \| \| 3124 \| \| 19 \| marginalisation.mp. \| \| 264 \| \| 20 \| SOCIAL MARGINALIZATION/ or marginalization.mp. or social isolation.mp. or Social Isolation/ \| \| 16295 \| \| 21 \| marginalised.mp. \| \| 613 \| \| 22 \| marginalized.mp. \| \| 2405 \| \| 23 \| Vulnerable Populations/ \| \| 8655 \| \| 24 \| (hard to reach adj1 (people or patient* or group* or population* or sample* or participant*)).tw. \| \| 593 \| \| 25 \| (seldom heard adj1 (people or patient* or group* or population* or sample* or participant*)).tw. \| \| 3 \| \| 26 \| ((vulnerabl* or exclude* or segregat* or discriminat* or disadvantag* or minority or disabled or poor or low-income or isolated or frail or polypharmacy or abused or maltreat or ill) adj1 (people or patient* or group* or population* or sample* or participant* or child* or older adult* or aged or elderly)).tw. \| \| 98256 \| \| 27 \| HOMELESS YOUTH/ or HOMELESS PERSONS/ or homeless*.mp. \| \| 10159 \| \| 28 \| substance user*.mp. or Alcoholism/ or Drug Users/ \| \| 75381 \| \| 29 \| sex worker*.mp. or Sex Workers/ \| \| 4639 \| \| 30 \| refugees.mp. or REFUGEES/ \| \| 9774 \| \| 31 \| "Transients and Migrants"/ or asylum-seeker*.mp. \| \| 11049 \| \| 32 \| indigenous people.mp. \| \| 1301 \| \| 33 \| minority ethnic groups.mp. \| \| 461 \| \| 34 \| Rare Diseases/ or rare disease*.mp. \| \| 22779 \| \| 35 \| care-leaver*.mp. \| \| 6 \| \| 36 \| looked-after child*.mp. \| \| 91 \| \| 37 \| Disabled Persons/ \| \| 38539 \| \| 38 \| ((economic* or social* or financial*) adj1 disadvantage*).tw. \| \| 3544 \| \| 39 \| (orphan adj1 (child or condition* or disease*)).tw. \| \| 673 \| \| 40 \| Child, Orphaned/ \| \| 628 \| \| 41 \| Deaf-Blind Disorders/ or Persons With Hearing Impairments/ or deafblind.mp. or Visually Impaired Persons/ \| \| 4451 \| \| 42 \| POVERTY/ or poverty.mp. \| \| 48835 \| \| 43 \| or/1-18 \| \| 190494 \| \| 44 \| or/19-42 \| \| 335111 \| \| 45 \| 43 and 44 \| \| 3903 \| \| 46 \| limit 45 to (abstracts and english language and yr="2000 -Current") \| \| 2679 \| \|  \| \| |  |
| --- | --- | --- | --- | --- | --- | --- | --- | --- | --- | --- | --- | --- | --- | --- | --- | --- | --- | --- | --- | --- | --- | --- | --- | --- | --- | --- | --- | --- | --- | --- | --- | --- | --- | --- | --- | --- | --- | --- | --- | --- | --- | --- | --- | --- | --- | --- | --- | --- | --- | --- | --- | --- | --- | --- | --- | --- | --- | --- | --- | --- | --- | --- | --- | --- | --- | --- | --- | --- | --- | --- | --- | --- | --- | --- | --- | --- | --- | --- | --- | --- | --- | --- | --- | --- | --- | --- | --- | --- | --- | --- | --- | --- | --- | --- | --- | --- | --- | --- | --- | --- | --- | --- | --- | --- | --- | --- | --- | --- | --- | --- | --- | --- | --- | --- | --- | --- | --- | --- | --- | --- | --- | --- | --- | --- | --- | --- | --- | --- | --- | --- | --- | --- | --- | --- | --- | --- | --- | --- | --- | --- | --- | --- | --- | --- | --- | --- | --- | --- | --- | --- | --- | --- | --- | --- | --- | --- | --- | --- | --- | --- | --- | --- | --- | --- | --- | --- | --- | --- | --- | --- | --- | --- | --- | --- | --- | --- | --- | --- | --- | --- | --- | --- | --- | --- | --- | --- | --- | --- | --- | --- | --- |
